# Regional variation of hysterectomy for benign uterine diseases in Switzerland

**DOI:** 10.1371/journal.pone.0233082

**Published:** 2020-05-14

**Authors:** Nina Stoller, Maria M. Wertli, Tabea M. Zaugg, Alan G. Haynes, Arnaud Chiolero, Nicolas Rodondi, Radoslaw Panczak, Drahomir Aujesky

**Affiliations:** 1 Department of General Internal Medicine, Inselspital, Bern University Hospital, University of Bern, Bern, Switzerland; 2 CTU Bern, University of Bern, Bern, Switzerland; 3 Institute of Primary Health Care (BIHAM), University of Bern, Bern, Switzerland; 4 Department of Epidemiology, Biostatistics, and Occupational Health, McGill University, Montreal, Canada; 5 Institute of Social and Preventive Medicine, University of Bern, Bern, Switzerland; 6 Queensland Centre for Population Research, School of Earth and Environmental Sciences, The University of Queensland, Brisbane, Australia; Universita degli Studi dell'Insubria, ITALY

## Abstract

**Background:**

Hysterectomy is the last treatment option for benign uterine diseases, and vaginal hysterectomy is preferred over more invasive techniques. We assessed the regional variation in hysterectomy rates for benign uterine diseases across Switzerland and explored potential determinants of variation.

**Methods:**

We conducted a population-based analysis using patient discharge data from all Swiss hospitals between 2013 and 2016. Hospital service areas (HSAs) for hysterectomies were derived by analyzing patient flows. We calculated age-standardized mean procedure rates and measures of regional variation (extremal quotient [EQ], highest divided by lowest rate) and systematic component of variation [SCV]). We estimated the reduction in the variance of crude hysterectomy rates across HSAs in multilevel regression models, with incremental adjustment for procedure year, age, cultural/socioeconomic factors, burden of disease, and density of gynecologists.

**Results:**

Overall, 40,211 hysterectomies from 54 HSAs were analyzed. The mean age-standardized hysterectomy rate was 298/100,000 women (range 186–456). While the variation in overall procedure rate was moderate (EQ 2.5, SCV 3.7), we found a very high procedure-specific variation (EQ vaginal 5.0, laparoscopic 6.3, abdominal 8.0; SCV vaginal 17.5, laparoscopic 11.2, abdominal 16.9). Adjusted for procedure year, demographic, cultural, and sociodemographic factors, a large share (64%) of the variance remained unexplained (vaginal 63%, laparoscopic 85%, abdominal 70%). The main determinants of variation were socioeconomic/cultural factors. Burden of disease and the density of gynecologists was not associated with procedure rates.

**Conclusions:**

Switzerland has a very high regional variation in vaginal, laparoscopic, and abdominal hysterectomy for benign uterine disease. After adjustment for potential determinants of variation including demographic factors, socioeconomic and cultural factors, burden of disease, and the density of gynecologists, two thirds of the variation remain unexplained.

## Background

Hysterectomy is one of the most common elective surgical procedures worldwide [1] and a generally accepted treatment for uterine cancer [2]. However, for benign uterine diseases, such as uterine fibroids (e.g., leiomyomas), endometriosis, abnormal uterine bleeding, and uterine prolapse, hysterectomy should be considered only when other treatment options fail [3–5]. A variety of non-surgical or minimally invasive treatment options are available and recommended by the gynecology societies depending on the underlying pathology. For example, in women with endometriosis, hysterectomy is not recommended unless pharmacological and minimally invasive treatment strategies fail to control symptoms and after family planning is completed [3–5]. Although the procedure is considered to be safe, complications may occur including infections (9–13%), venous thromboembolism (1–12%), and genitourinary and gastrointestinal tract injuries (1–2%) [6] depending on the specific procedure performed [7]. Moreover, guidelines recommend vaginal hysterectomy as the first choice due to fewer intra- and postoperative complications [8,9], shorter operation and hospitalization times [5,10,11], and lower healthcare costs [10]. When vaginal hysterectomy is not possible, the less invasive laparoscopic hysterectomy is preferable over the more invasive abdominal hysterectomy [3,11], due to fewer complications [7]. Abdominal hysterectomy is recommended for extrauterine disease, and when uterus size precludes other procedures [3,5,11].

Despite recommendations to use hysterectomy restrictively [3,5,11], the procedure rates and types vary substantially between and within countries [1]. While variations in the use of elective, preference-sensitive procedures can be partially attributed to differently structured healthcare systems and cultural differences [12,13], variation in hysterectomy and the use of different procedures remain poorly understood.

**Fig 1:**
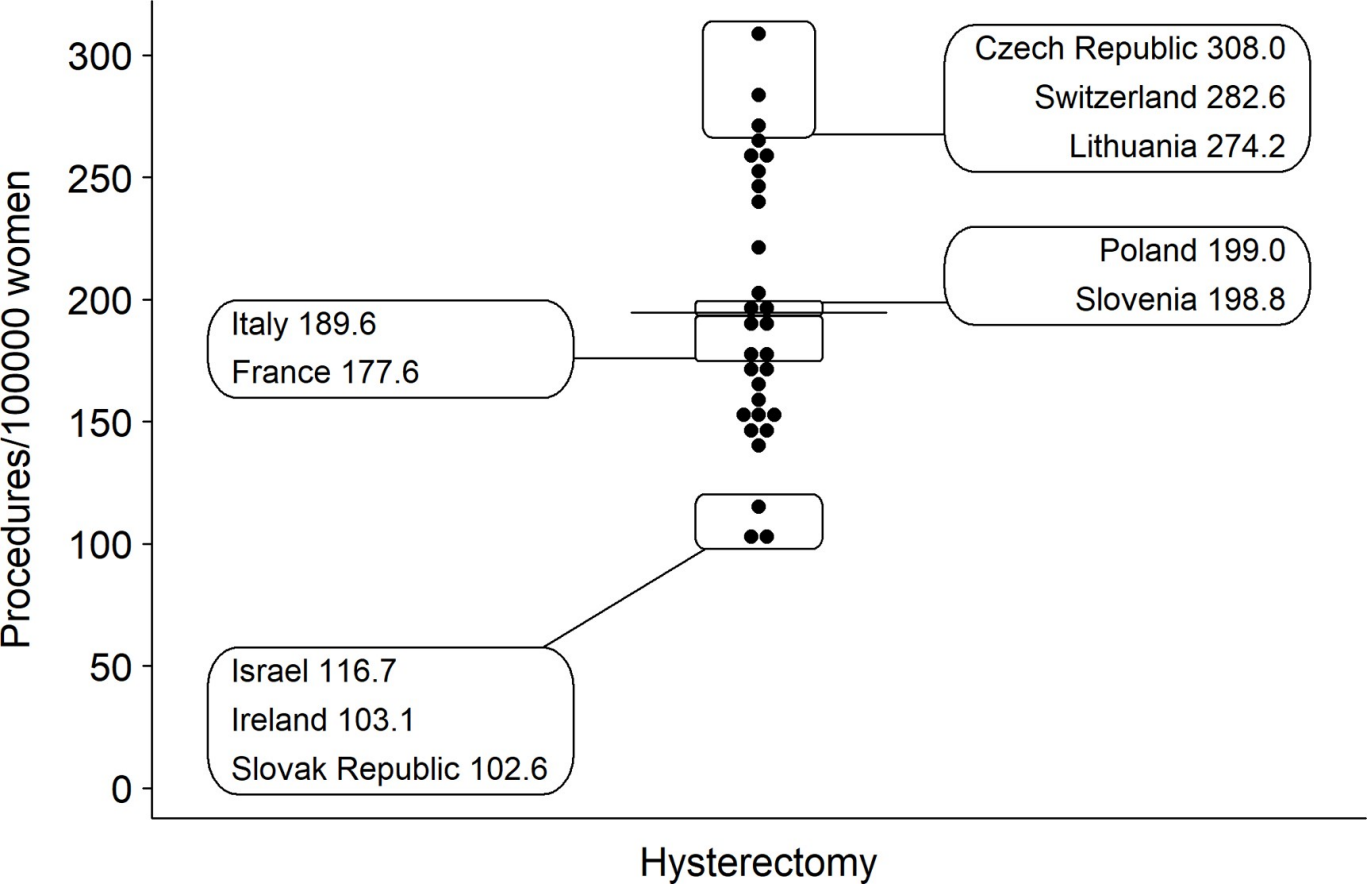
Comparison of crude hysterectomy rates across OECD countries in 2015 [1]. Horizontal line represents the OECD average rate.

Switzerland has one of the highest hysterectomy rates (**Fig 1**) of all OECD countries, with an average of 283 procedures per 100,000 women in 2016 [1]. We aimed therefore to examine (1) the regional variation and (2) factors that drive overall and procedure-specific hysterectomy rates for benign uterine diseases across Switzerland from 2013–2016 and to explore potential determinants of variation.

## Methods

### Data sources

A population-based, small area analysis based on routinely collected patient discharge data from all public and private Swiss acute care hospitals and census data was conducted for calendar years 2013–2016 [14]. Swiss hospitals are legally obligated to provide the Swiss Federal Statistical Office (SFSO) with an anonymized, standardized data set for each hospital discharge. These data are then combined and centrally stored in the Swiss Hospital Discharge Masterfile hosted at the SFSO. Recorded variables include patient age, sex, nationality, insurance status, the type of admission (emergency vs. elective), up to 100 procedure codes based on the Swiss Classification of Operations (CHOP, an adaptation of the U.S. ICD-9-CM volume 3 procedure classification) [15], and up to 50 diagnostic codes based on the International Classification of Diseases, 10^th^ revision, German Modification (ICD-10-GM). Further, the area of patient residence and hospital location within one of 705 Swiss MedStat regions are recorded. MedStat regions represent Swiss statistical regions based on aggregated ZIP-codes [16]. The SFSO reviews data integrity and completeness by means of a specifically designed software [17]. Since 2012, the Swiss Hospital Discharge Masterfile covers 100% of discharges, and data completeness for CHOP codes used in this analysis was high [18].

We used Swiss National Cohort data [19] to define the language (Swiss German, Romance [French or Italian]) and data from the SFSO spatial planning from 2014 to determine the level of urbanization (urban, peri-urban, rural area) for each MedStat region. The level of urbanization was based on the Degree of urbanization (DEGURBA) classification used by the European Union [20]. We abstracted measures of socioeconomic status (neighborhood information on rent, education, occupation, and crowding) from 2000 Swiss census data [21]. Finally, we obtained the density and average time since graduation of gynecologists per MedStat region for calendar year 2014 from the registry of physicians affiliated with the Swiss Medical Association (FMH). Our study was based on anonymized administrative data only and was thus exempted from ethics committee approval according to the Swiss Human Research Act.

### Derivation of hysterectomy-specific hospital service areas

Switzerland has compulsory basic health insurance coverage, with voluntary semiprivate and private insurance plans covering additional medical services. Although Swiss hospital care is primarily organized based on 26 geographic regions, the cantons, patients may utilize hospital services outside their canton of residence and the use of cantons as a unit of observation may skew procedure rates. We therefore used a fully automated method to generate reproducible general hospital service areas (HSAs) using all patients discharge data from calendar years 2013–2016 [22]. The process to derive HSAs is based on a valid method described by the pioneers of health services research [23] and has been previously described [22]. In a first step, we identified 4,105,885 discharges of patients aged ≥18 years living in Switzerland from 155 Swiss acute care hospitals for calendar years 2013–2016 (**S1 Fig**). We then analyzed patient flows and assigned MedStat regions from which the highest proportion of residents was discharged to the same HSA (plurality rule) [24]. HSAs with <50% of the patients being treated within the same HSA or <10 procedures overall were merged with the neighboring HSA which received most discharges until >50% and ≥10 procedures were performed within each HSA. This process yielded 63 general HSAs. In a second step, we identified patient discharges with specific CHOP codes for vaginal (codes 68.43, 68.59, 68.63, 68.79), laparoscopic (68.31, 68.41, 68.44, 68.51, 68.61, 68.64, 68.71), and abdominal hysterectomies (68.30, 68.32, 68.39, 68.40, 68.42, 68.49, 68.62, 68.69) from all Swiss acute care hospitals for calendar years 2013–2016 using the Swiss Hospital Discharge Masterfile. As hysterectomies are not performed in every hospital, we further collapsed the 63 general HSAs into 54 hysterectomy-specific HSAs. We then drew visual maps of the 54 final HSAs using Geographical Information System (GIS)-compatible vector files.

### Study population

Overall, we identified 46,897 discharges with specific codes for hysterectomy who had a MedStat region of residence code. After delineating the HSAs, we excluded all discharges related to emergencies (i.e., hysterectomy performed within the first 12 hours of the hospitalization; n = 1,369), and hysterectomies related to tumor (n = 5,220) or obstetric surgery (n = 97, **S1 Table**), leaving a final study population of 40,211 patient discharges with hysterectomy (**S1 Fig**). Discharges with more than one procedure were assigned to the most invasive procedure. Laparoscopically assisted vaginal hysterectomy was assigned to the laparoscopic hysterectomy group.

### Measures of variation

We planned to examine the association between potentially influential factors and (1) overall hysterectomy and (2) procedure-specific interventions (abdominal, vaginal, and laparoscopic). We calculated age-standardized hysterectomy procedure rates per 100,000 women for each HSA using procedure counts and 2013–2016 census data for the female Swiss population [25]. We used direct standardization with age bands of 18–49, 50–54, 55–60, (…), 75–80 and ≥80 years. To examine the variation in procedure rates across hysterectomy-specific HSAs, we determined the extremal quotient (EQ), which is the highest divided by the lowest procedure rate. While the EQ is an intuitive measure of variation, it is prone to distortion by extreme values [12]. We also calculated the coefficient of variation (CV), i.e., the ratio of the standard deviation of the procedures rates to the mean rate, the systematic component of variation (SCV), and the Empirical Bayes (EB) statistic [12,26,27]. Although less intuitive, the SCV represents the non-random part of the variation in procedure rates while reducing the effect of extreme values [12,26,28]. An SCV of >5 is considered indicative of a high variation and an SCV of >10 of a very high variation [12,28]. The EB statistic is another measure of the non-random part of the variation using the Penalized Quasi Likelihood method which is based on the assumption that the log-relative risks are normally and identically distributed [27]. While both the SCV and the EB statistics assess the non-random variation with a result of zero indicating no variation across HSAs, the EB statistic is not influenced by the procedure rate [27].

### Determinants of variation

Differences in illness incidences and socioeconomic factors are possible and legitimate causes of variation [12]. We therefore explored four domains that could influence the rates: demographics (age), cultural and socioeconomic factors (language region, level of urbanization, Swiss neighborhood index of socioeconomic position [SSEP], insurance status, and Swiss citizenship), population health (burden of disease), and supply factors (physician density and average time since graduation). As a proxy for the population burden of disease, we calculated age-standardized incidence rates of hip fractures, colon or lung cancer treated surgically, acute myocardial infarctions, or strokes for each HSA (**S2 Table**), as differences in these disease rates are likely to reflect true regional differences in burden of disease rather than differences in coding intensity or supply factors [29,30]. We used an adapted form of the Degree of Urbanization (DEGURBA) classification [20] by the European statistical office to assign the level of urbanization with the most inhabitants for each HSA. Urbanicity is classified into 3 three area types: 1) urban areas: cities (densely populated areas) with at least 50% of the population living in urban centers, 2) peri-urban areas: towns and suburbs (intermediate density areas) with less than 50% of the population living in rural grid cells and less than 50% of the population lives in urban centers, and 3) rural areas (thinly populated areas) with more than 50% of the population living in rural grid cells. We attributed the language to a given HSA that was spoken by most people living within the HSA. The socioeconomic status of each HSA was calculated using the mean SSEP of all neighborhoods within an HSA [21]. The SSEP consists of four domains (median rent/m2, proportion of households led by a person with no/low education, proportion headed by a person in manual/unskilled occupation, and mean crowding, all on the neighborhood level). The SSEP varies between zero (worst) and 100 (best) and correlates well with mortality [31]. The percentage of discharges with (semi)private insurance status and Swiss citizenship was used as an additional measure of the socioeconomic status of each HSA. The density of gynecologists and the average time since graduation were used as supply measures. As gynecology training usually is completed within 10 years since graduation in Switzerland, the time since graduation serves as a proxy for the gynecologists’ professional experience and may reflect the surgical methods that were taught in teaching hospitals at this time.

### Statistical analyses

We used progressively adjusted multilevel Poisson regression with a log link to model the procedure rates in each HSA using age bands of 18–49, 50–54, 55–60, (…), 75–80, and ≥80 years. HSA was included as a random intercept in all models. In a progressive approach, model 1 was adjusted for the calendar year of the procedure, model 2 in addition for demographics, model 3 added socioeconomic and cultural factor, model 4 regional health, and model 5 supply factors. Variables included in the model were chosen *a priori* as we expected them to influence the rates. The models were used in three ways: 1) to assess to which extent explanatory factors explain hysterectomy rates in Switzerland, 2) to assess the variance explained by the domains defined previously, and 3) to calculate intervention rates per 100,000 women per HSA. For the first, we expressed the effect of explanatory factors on hysterectomy rates as incidence rate ratios (IRRs), defined as the hysterectomy rate in the defined category (e.g., Romance language region) relative to the estimated hysterectomy rate in the reference category (e.g., Swiss German language region). For the second use, we determined the percentage reduction in procedure variation across the 54 HSAs by examining the variance of the random intercept relative to model 1. We considered the residual, unexplained variation of the fully adjusted model a proxy for unwarranted variation. For the third use, we used the models to predict rates in each HSA. Sets of models were created for overall rates as well as abdominal, laparoscopic, and vaginal hysterectomies. Statistical modeling was performed using Stata version 15.1 (StataCorp, College Station, TX, USA). HSAs were delineated and maps drawn using the R statistical software, version 3.4.2 [32].

## Results

### Characteristics of hysterectomy-specific HSAs and the study population

The median population size per HSA was 46,617 women (interquartile range [IQR] 22,853–85,869), with a median population density of 132 women/km^2^ (IQR 45–235), a mean SSEP of 62 points (standard deviation [SD] 6), and a mean density of gynecologists of 16.7 (SD 5) per 10,000 women. Gynecologists’ median of the average time since graduation was 23 years (IQR 21–26). Overall, 38 HSAs were located in the Swiss German and 16 in Romance (12 French and 4 Italian) language regions.

Of the 40,211 women discharged after hysterectomy, 11,691 (29%) underwent vaginal, 20,185 (50%) laparoscopic, and 8,288 (21%) abdominal hysterectomy. In laparoscopic hysterectomy, robotic assisted procedures were performed in less than 6% of procedures. The majority of women were 40–60 years old (26,945 women, 67%), Swiss citizens (81%), and had a general insurance status (74%, **Table 1**).

**Table 1:** Characteristics of the study population undergoing abdominal, laparoscopic, or vaginal hysterectomy during calendar years 2013–2016.

	Total (N = 40,211)	Vaginal (N = 11,691)	Laparoscopic (N = 20,185)	Abdominal (N = 8,288)
*N (%)*				
Age [years]				
18–49	21,985 (55)	3,958 (34)	13,027 (64)	4,976 (60)
50–59	9,208 (23)	2,481 (21)	4,669 (23)	2,044 (25)
60–69	4,695 (12)	2,439 (21)	1,575 (8)	676 (8)
70–79	3,301 (8)	2,066 (18)	780 (4)	451 (5)
≥80	1,022 (3)	747 (6)	134 (1)	141 (2)
Insurance status				
General	29,725 (74)	8,712 (75)	14,690 (73)	6,308 (76)
(Semi)private	10,486 (26)	2,979 (25)	5,495 (27)	1,980 (24)
Citizenship				
Swiss	32,495 (81)	9,978 (85)	15,869 (79)	6,613 (80)
Non-Swiss	7,716 (19)	1,713 (15)	4,316 (21)	1,675 (20)

### Variation in procedure rates across HSAs

**Fig 2:**
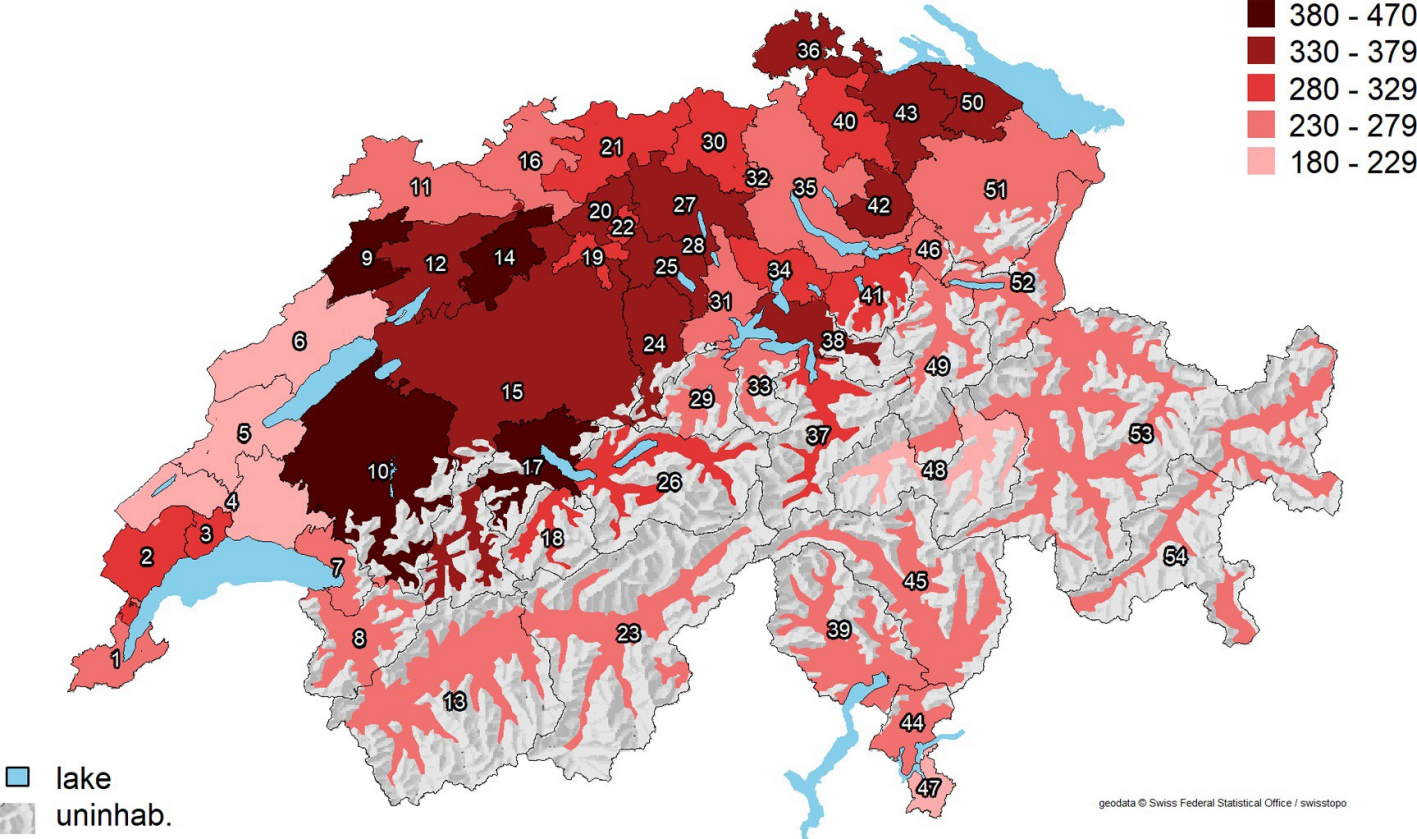
Age-standardized annual hysterectomy rates (per 100,000 women) across 54 Swiss HSAs. Abbreviations: uninhab. = uninhabited area; HSA = hospital service area. Average predicted hysterectomy rates for each HSA are shown as red-scale categories per 100,000 women. Reprinted from the Federal Office of Topography swisstopo, Switzerland (https://shop.swisstopo.admin.ch/en/products/maps/overview/relief and shape files derived from postcode-level shape file used to create map of Switzerland, e.g., https://www.geocat.ch/geonetwork/srv/ger/md.viewer#/full_view/973cd117-f1ed-481) under a CC BY license, with permission from Alexandra Frank, original copyright 2006.

The mean age-standardized overall procedure rate was 298 (range 186–456) per 100,000 women (**Fig 2**), as opposed to an average of 191 hysterectomies per 100,000 women performed in OECD countries in 2016 [1]. The EQ in Switzerland was 2.5, the CV 0.2, the EB 0.03, and the SCV 3.7 (**Table 2**), indicating a moderate variation across HSAs. After full adjustment for demographics, cultural and socioeconomic factors, burden of disease, and density of gynecologists, the predicted hysterectomy rates varied between 208 and 407 per 100,000 women, of which two were above 380 (HSA number 10 and 14) and four below 230 per 100,000 women (HSAs 4–6 and 48).

**Table 2:** Measures of variation in procedure rates across hysterectomy-specific HSAs.

	EQ	CV	SCV	EB
Overall	2.5	0.2	3.7	0.03
Vaginal	5.0	0.4	17.5	0.2
Laparoscopic	6.4	0.4	11.2	0.1
Abdominal	8.0	0.4	16.9	0.1

Abbreviations: EQ = extremal quotient; CV = coefficient of variation; SVC = systematic component of variation; EB = empirical Bayes

**Fig 3:**
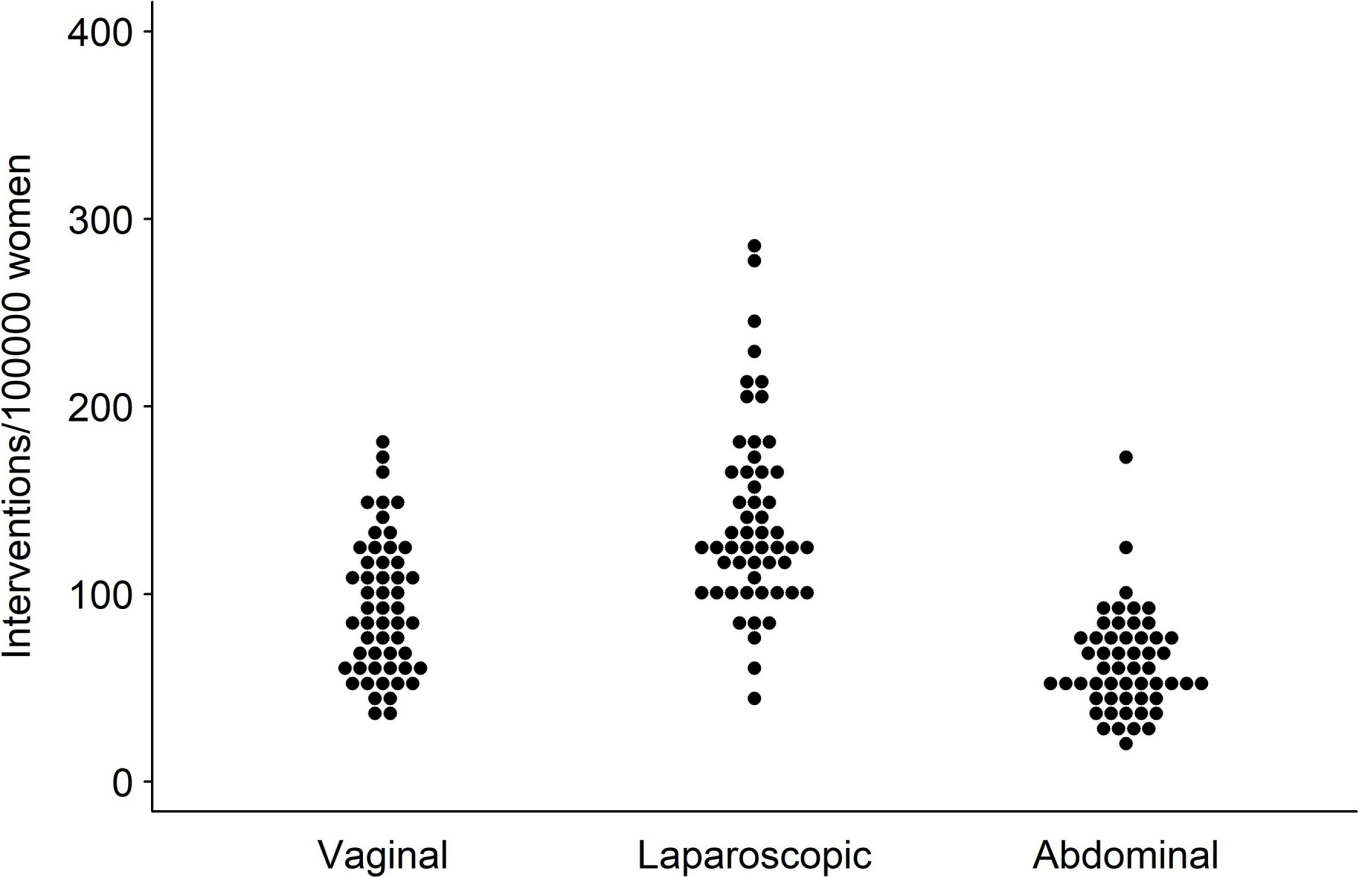
Variation in age-standardized vaginal, laparoscopic, and abdominal hysterectomy rates across 54 Swiss HSAs. Each dot represents one HSA.

The age-standardized procedure rates for vaginal hysterectomy was 94 (35–178) per 100,000 women. The EQ was 5.0, the CV 0.4, the EB 0.2, and the SCV 17.5 (**Table 2**), indicating a very high variation. The mean age-standardized rates for laparoscopic hysterectomy was 140 (45–289) and for abdominal hysterectomy 63 (21–172) per 100,000 women. Both laparoscopic (EQ 6.4, CV 0.4, EB 0.1, and SCV 11.2, **Table 2**) and abdominal hysterectomy rates (EQ 8.0, CV 0.4, EB 0.1, and SCV 16.9, **Table 2**) showed a very high regional variation. **Fig 3**depicts the variation in age-standardized vaginal, laparoscopic, and abdominal hysterectomy rates across HSAs. Detailed age-standardized hysterectomy rates for each HSA are shown in the **S3 Table**.

### Determinants of variation in hysterectomy rates

Between 2013 and 2016, overall hysterectomy rates decreased by 6% (IRR 0.94, 95% confidence interval [CI]: 0.91–0.97, **Table 3**). Cultural and socioeconomic factors were the main determinants of procedure variation across HSAs. Hysterectomy rates were highest in women aged 50–54 years. Residence in a Romance language region was associated with an 11% lower hysterectomy rate (IRR 0.89; 95%CI: 0.78–0.99 compared to the Swiss German language region). Compared to the year-adjusted model, adjustment for age resulted in a 4% reduction in the variance of procedure rates and further adjustment for cultural/socioeconomic factors resulted in an additional 29% reduction in variance. Adjustment for health and supply factors explained 2% of the variance (total variance unexplained by the full model 64%).

**Table 3:** Determinants of variance in the incidence rates of hysterectomy across 54 Swiss HSAs.

		Model 1*	Model 2†	Model 3‡	Model 4#	Model 5&
		Incidence rate ratio (95%CI)§				
**Case year**	2013	Reference	Reference	Reference	Reference	Reference
	2014	0.98 (0.96–1.01)	0.98 (0.96–1.01)	0.99 (0.96–1.01)	0.98 (0.96–1.01)	0.98 (0.96–1.01)
	**2015**	**0.96 (0.93**–**0.98)**	**0.96 (0.93**–**0.98)**	**0.96 (0.93**–**0.99)**	**0.95 (0.92**–**0.98)**	**0.95 (0.92**–**0.98)**
	**2016**	**0.94 (0.91**–**0.97)**	**0.94 (0.91**–**0.97)**	**0.94 (0.92**–**0.97)**	**0.94 (0.91**–**0.97)**	**0.94 (0.91**–**0.97)**
**Age**	**18**–**49**		**0.62 (0.61**–**0.64)**	**0.62 (0.61**–**0.64)**	**0.62 (0.61**–**0.64)**	**0.62 (0.61**–**0.64)**
	50–54		Reference	Reference	Reference	Reference
	**55**–**59**		**0.54 (0.52**–**0.57)**	**0.54 (0.52**–**0.57)**	**0.54 (0.52**–**0.57)**	**0.54 (0.52**–**0.57)**
	**60**–**64**		**0.51 (0.48**–**0.53)**	**0.51 (0.48**–**0.53)**	**0.51 (0.48**–**0.53)**	**0.51 (0.48**–**0.53)**
	**65**–**69**		**0.54 (0.51**–**0.56)**	**0.54 (0.51**–**0.56)**	**0.54 (0.51**–**0.56)**	**0.54 (0.51**–**0.56)**
	**70**–**74**		**0.52 (0.49**–**0.54)**	**0.52 (0.49**–**0.54)**	**0.52 (0.49**–**0.54)**	**0.52 (0.49**–**0.54)**
	**75**–**79**		**0.46 (0.44**–**0.49)**	**0.46 (0.44**–**0.49)**	**0.46 (0.44**–**0.49)**	**0.46 (0.44**–**0.49)**
	≥**80**		**0.20 (0.19**–**0.21)**	**0.20 (0.19**–**0.21)**	**0.20 (0.19**–**0.21)**	**0.20 (0.19**–**0.21)**
**Language region**	Swiss German			Reference	Reference	Reference
	**Romance**			0.89 (0.80–1.00)	**0.89 (0.80**–**0.99)**	**0.89 (0.80**–**0.99)**
Level of urbanization	Urban			Reference	Reference	Reference
	Peri-urban			1.19 (1.04–1.37)	1.18 (1.02–1.35)	1.14 (0.98–1.33)
	Rural			1.21 (0.99–1.47)	1.19 (0.98–1.45)	1.15 (0.93–1.43)
Mean SSEP (per 10 units)				1.03 (0.89–1.19)	1.02 (0.89–1.17)	1.04 (0.91–1.20)
(Semi)private insurance status (per 10% change)				1.03 (0.95–1.12)	1.04 (0.96–1.13)	1.02 (0.94–1.11)
Swiss citizenship (per 10% change)				1.02 (0.94–1.10)	1.02 (0.95–1.11)	1.02 (0.95–1.11)
Burden of disease (per 1000 women)**					1.08 (0.96–1.22)	1.08 (0.96–1.22)
Gynecologists (per 1/10,000 change)***						1.00 (0.98–1.01)
Average time since graduation (per 5 years)						1.05 (0.97–1.14)
Remaining variance from the model (%)††			96.1	67.6	66.1	63.9

Abbreviations: CI = confidence interval; SSEP = Swiss neighborhood index of socioeconomic position. Results in **bold** indicate a statistically significant effect.

*Model 1: adjusted for the year of the procedure.

†Model 2: additional adjustment for age.

‡Model 3: additional adjustment for language region, socioeconomic factors (level of urbanization, SSEP, insurance status, and Swiss citizenship).

#Model 4: additional adjustment for burden of disease.

&Model 5: additional adjustment for the density of gynecologists and the average time since graduation.

§Hysterectomy rate in the defined category relative to the hysterectomy rate in the reference category. For instance, an incidence rate ratio of 0.95 indicates a 5% lower hysterectomy rate in Romance language regions than in Swiss German language regions.

**Sum of age-standardized incidence rates per 1000 women for hip fracture, colon or lung cancer treated surgically, acute myocardial infarction, and stroke. The IRR is the increase (or decrease in rates) when the burden of disease changes from e.g. 3 women with a comorbidity per 1000 to 4 women with a comorbidity per 1000 women.

***Density of gynecologists per 10,000 women. The IRR is the increase (or decrease) in rates when the density of gynecologists changes from e.g. 2 gynecologists per 10,000 persons to 3 per 10,000 women.

††Expresses the variance in hysterectomy rates from the average rate.

### Determinants of variation in procedure-specific rates

Whereas vaginal hysterectomy rates decreased by 24% (IRR 0.76, 95%CI: 0.71–0.80, **Table 4**) and abdominal hysterectomies by 34% (IRR 0.66, 95%CI: 0.62–0.71), the laparoscopic hysterectomy rates increased by 23% (IRR 1.23, 95%CI: 1.18–1.29) during 2013–2016. The proportion of laparoscopic interventions with robotic codes per year was low (4% in 2013, 5% in 2014, 6% in 2015, and 5% in 2016). Cultural/socioeconomic factors remained the most relevant determinants for all three procedures. For vaginal hysterectomies, age was associated with higher procedure rates (IRR 1.37, 95%CI: 1.27–1.48 for women aged 70–74 compared to women aged 50–54 years). Residence in a Romance language region was associated with a 40% lower vaginal hysterectomy rate (IRR 0.60, 95%CI: 0.48–0.75) but not with laparoscopic and abdominal hysterectomy rates. Residence in a rural area was associated with a 59% higher abdominal hysterectomy rate (IRR 1.59, 95%CI: 1.01–2.49) compared to urban and peri-urban areas. An increased burden of disease resulted in 33% higher abdominal hysterectomy rate (IRR 1.33, 95%CI: 1.02–1.72), with no significant effect on the other procedures. Adjustment for cultural/socioeconomic factors explained 36% of the variance in vaginal hysterectomy rates, 11% in laparoscopic, and 15% in abdominal hysterectomy rates. Additional adjustment for health and supply factors resulted in no or minimal further reduction in the variance in vaginal and laparoscopic hysterectomy. Adjustment for health explained 7% and supply factors 3% of the variance in abdominal hysterectomies. Gynecologists’ average time since graduation was associated with a 21% higher abdominal hysterectomy rate (IRR 1.21, 95%CI: 1.02–1.41). Sixty-three percent of the total variance in vaginal, 85% in laparoscopic and 70% in abdominal hysterectomy remained unexplained.

**Table 4:** Fully adjusted models for procedure-specific hysterectomy rates across 54 Swiss HSAs.

		Vaginal	Laparoscopic	Abdominal
		Incidence rate ratio (95%CI)§		
**Case year**	2013	Reference	Reference	Reference
	2014	**0.95 (0.90**–**1.00)**	**1.09 (1.05**–**1.14)**	**0.84 (0.79**–**0.89)**
	2015	**0.86 (0.81**–**0.91)**	**1.13 (1.08**–**1.18)**	**0.76 (0.72**–**0.81)**
	2016	**0.76 (0.71**–**0.80)**	**1.23 (1.18**–**1.29)**	**0.66 (0.62**–**0.71)**
**Age**	18–49	**0.50 (0.47**–**0.53)**	**0.69 (0.67**–**0.72)**	**0.59 (0.56**–**0.63)**
	50–54	Reference	Reference	Reference
	55–59	**0.87 (0.80**–**0.94)**	**0.46 (0.43**–**0.49)**	**0.42 (0.38**–**0.47)**
	60–64	**1.11 (1.03**–**1.20)**	**0.34 (0.32**–**0.37)**	**0.30 (0.27**–**0.34)**
	65–69	**1.29 (1.19**–**1.39)**	**0.31 (0.29**–**0.34)**	**0.32 (0.29**–**0.36)**
	70–74	**1.37 (1.27**–**1.48)**	**0.25 (0.23**–**0.28)**	**0.29 (0.26**–**0.33)**
	75–79	**1.36 (1.25**–**1.48)**	**0.17 (0.15**–**0.20)**	**0.26 (0.22**–**0.30)**
	≥80	**0.65 (0.60**–**0.71)**	**0.05 (0.04**–**0.06)**	**0.11 (0.10**–**0.13)**
**Language region**	Swiss German	Reference	Reference	Reference
	**Romance**	**0.60 (0.48**–**0.75)**	1.10 (0.89–1.36)	1.05 (0.84–1.31)
**Level of urbanization**	Urban	Reference	Reference	Reference
	Peri-urban	1.14 (0.83–1.57)	1.12 (0.82–1.53)	1.30 (0.94–1.78)
	**Rural**	1.38 (0.88–2.18)	0.87 (0.56–1.36)	**1.59 (1.01–2.49)**
Mean SSEP (per 10 units)		0.82 (0.61–1.10)	1.16 (0.87–1.54)	0.99 (0.74–1.33)
(Semi)private insurance status (per 10% change)		1.09 (0.92–1.29)	1.06 (0.90–1.24)	0.97 (0.82–1.15)
Swiss citizenship (per 10% change)		0.92 (0.78–1.08)	1.11 (0.98–1.25)	1.02 (0.87–1.20)
Burden of disease (per 1000 women)**		1.11 (0.89–1.38)	0.92 (0.77–1.10)	**1.33 (1.02**–**1.72)**
Gynecologists (per 1/10,000 change)***		1.00 (0.98–1.02)	0.99 (0.96–1.01)	1.02 (0.99–1.04)
Average time since graduation (per 5 years)		1.11 (0.94–1.31)	0.99 (0.84–1.18)	**1.21 (1.02–1.41)**
Remaining variance from the fully adjusted model (%)††		63.3	85.3	70.4

Abbreviations: CI = confidence interval; SSEP = Swiss neighborhood index of socioeconomic position. Results in **bold** indicate a statistically significant effect.

§Hysterectomy rate in the defined category relative to the hysterectomy rate in the reference category.

**Sum of age-standardized incidence rates per 1000 women for hip fracture, colon or lung cancer treated surgically, acute myocardial infarction, and stroke. The IRR is the increase (or decrease in rates) when the burden of disease changes from e.g. 3 women with a comorbidity per 1000 to 4 women with a comorbidity per 1000 women.

***Density of gynecologists per 10,000 women. The IRR is the increase (or decrease) in rates when the density of gynecologists changes from e.g. 2 gynecologists per 10,000 persons to 3 per 10,000 women.

††Expresses the variance in hysterectomy rates from the average national rate.

## Discussion

We found a moderate variation in overall and a very high variation in vaginal, laparoscopic, and abdominal hysterectomy rates for benign uterine disease across 54 Swiss HSAs. Only about one third of the variation in overall procedure rates was explained by differences in age, language, and socioeconomic factors.

While a moderate decrease in overall hysterectomy rates by 16% [1] was observed in Switzerland between 2002 and 2016 (6% from 2013 to 2016), the rates decreased by more than 30% in France and the USA [33] and by more than 50% in Finland during the same time period [1]. Our data demonstrate that vaginal and abdominal hysterectomy is increasingly replaced by laparoscopic hysterectomy, a phenomenon that has also been observed in other countries [7,34–37]. While the decrease in invasive abdominal procedures may be desirable due to fewer complications [7], the substitution of vaginal hysterectomies by laparoscopic procedures is not in agreement with guideline recommendations [3–5,11]. Compared to vaginal hysterectomy, laparoscopic hysterectomy is associated with a 6-fold higher vaginal cuff dehiscence and an almost 4-fold higher conversion rate in laparotomy, more blood loss and visceral injuries, a longer duration of surgery and hospital stay, and lower costs [10,35,38].

Our results indicate that the uptake of recommendations to use vaginal hysterectomy as the first choice for benign uterine disease [3–5,11] was very heterogeneous among Swiss gynecologists and may reflect differing physicians’ beliefs on its indication and efficacy [26,39–41]. As reimbursement rates for hysterectomy are similar across Swiss regions and do not depend on the type of intervention used, differing financial incentives are unlikely to explain differences in procedure rates and types. Some gynecologists may consider laparoscopic hysterectomy as a more advanced technique because of a better visualization of the operation field, or lack experience leading to reluctance to perform vaginal hysterectomy [41–43].

Factors considered in the choice of a procedure include the size of the uterus and the vagina [11,44]. In very large uteri, laparoscopic procedures may not be feasible, and the advantages and disadvantages of minimally invasive approaches using morcellation techniques should be weighed against the increased risk of complication in abdominal hysterectomy [11]. Although uncommon, the unintended morcellation and removal of uterine cancer may result in the spread of tumor cells [2]. In small uteri, less invasive procedures and pharmacological treatments may also be effective and should be considered before more invasive treatment options [11].

While women at the age of menopause had the highest abdominal and laparoscopic hysterectomy rates [45–47], women aged 70–79 years had the highest rate of vaginal hysterectomy, which may be due to a higher prevalence of pelvic organ prolapse and comorbid conditions in the elderly [48]. We also observed higher hysterectomy rates in Swiss German-speaking compared to Romance-speaking areas. In 1984, a public media campaign was conducted in the Italian-speaking Canton of Ticino to reduce hysterectomy rates [49]. As a result, the annual hysterectomy rate dropped by 26% during the following year, while an 1% increase was observed in a reference area without media campaign (Swiss German-speaking Canton of Berne) [49]. Other microcultural factors may also drive health care use in Switzerland [50,51]. For instance, residents of Swiss Romance language regions were shown to consult specialists more frequently [50] and to have higher per-capita healthcare costs [52] than residents in Swiss German language regions. However, our results and others suggest that the higher healthcare use in Romance language regions may not necessarily extend to elective invasive procedures, such as gynecological and orthopedic interventions [14]. Interestingly, the lower hysterectomy rates among Romance-speaking HSAs appear to be driven by a lower rate of vaginal procedures, with slightly higher rates of abdominal and laparoscopic hysterectomy compared to Swiss German HSAs.

Hysterectomy rates were similar across different levels of urbanization except for abdominal hysterectomy which was more often performed in rural areas. In contrast, in Australia hysterectomy rates were higher in peri-urban areas than in major cities or rural areas [53]. Urbanicity-related differences in procedure rates are difficult to explain but may be due to differences in the availability of alternative treatments, the needs and preferences of women [53], lack of training/experience in vaginal or laparoscopic procedures in rural areas [54,55], or family physicians’ referral practices [53].

Women with an increased preoperative risk were more likely to undergo abdominal hysterectomy and less likely to undergo minimally invasive procedures [56]. In HSAs with higher disease burden, higher abdominal hysterectomy rates were observed in this study. One explanation may be that women with more comorbidity are only scheduled for hysterectomy when they have a more advanced and serious disease. Given the higher complication rate of abdominal hysterectomy [10], further research is required to investigate the reasons why gynecologists are choosing this procedure in sicker patients [53]. In contrast to prior studies [53,57], we did not find higher hysterectomy rates in patients with private insurance or areas with a higher density of gynecologists. Training and personal experience may explain the higher abdominal hysterectomy rates in areas which have a gynecologist workforce that is longer in clinical practice [41].

In our study, only about one third of the variation in hysterectomy across Swiss HSAs was explained by differences in demographic, cultural, and socioeconomic factors. While we cannot entirely exclude the possibility that regional variations are at least partially due to differences in the prevalence/severity of benign uterine disease or patient preferences, the residual variation in procedure rates is much more likely due to local differences in physicians’ attitudes towards performing hysterectomy and the choice of the specific procedure [26,39–41].

Our work has potential limitations. First, we did not have data on gynecologic disease prevalence on a regional level (denominator). Thus, we could not examine whether regional differences in gynecologic disease prevalences drive regional procedure variation. However, it is highly unlikely that gynecologic diseases differ across geographically close regions. While we had a set of diagnostic codes for each discharge, coded diagnoses may not represent the primary indication for the procedure, and the disease prevalence in women undergoing hysterectomy may be biased due to diagnostic underreporting [18]. Further, SFSO data only cover inpatient procedures and therefore, we were not able to analyze procedures mainly done on an outpatient basis. While we found an association between procedure rates and determinants, we cannot infer causality. Relatedly, adjustment for ecological variables on a population level (i.e., SSEP, citizenship, and burden of disease) includes a risk of ecological fallacy by drawing conclusions about the behavior of individuals based on population level parameters [58]. As our smallest unit of analysis was the MedStat region (Swiss statistical regions based on aggregated ZIP-codes) and not individual hospitals and small and high volume hospitals could be within the same MedStat region, we could not examine the effect of hospital volume on procedure rates and types. Finally, our method of delineating HSAs does not yield homogenous regions in terms of size or population. It is possible, although difficult to confirm, that this could introduce bias due to HSAs representing varying degrees of aggregation and differing population sizes.

In conclusion, Switzerland has high overall hysterectomy rates for benign uterine disease, with a very high regional variation in vaginal, laparoscopic, and abdominal hysterectomy. While several cultural, and socioeconomic factors were associated with procedure rates, two thirds of the procedural variation remained unexplained and most likely represents differing physician attitudes towards hysterectomy and procedure choice rather than differences in patient need and preferences.

## Supporting information

S1 Table Overview of diagnostic codes used for exclusion. (DOCX)

S2 Table Overview of codes to define comorbidity burden. (DOCX)

S3 Table Crude- and age-standardized procedure-specific hysterectomy rates. (DOCX)

S1 Fig Study flow chart. (TIF)

S2 Fig Fully adjusted vaginal, laparoscopic, and abdominal hysterectomy rates. Abbreviations: uninhab. = uninhabited area, HSA = Health Service Area. Hysterectomy rates/100,000 women/per HSA (No. shown within HSA). Reprinted from the Federal Office of Topography swisstopo, Switzerland (https://shop.swisstopo.admin.ch/en/products/maps/overview/relief and shape files derived from postcode-level shape file used to create map of Switzerland, e.g., https://www.geocat.ch/geonetwork/srv/ger/md.viewer#/full_view/973cd117-f1ed-481) under a CC BY license, with permission from Alexandra Frank, original copyright 2006.
(TIFF)

S1 File STROBE statement. (DOC)
