# Supplementary material for: Regional variation of hysterectomy for benign uterine diseases in Switzerland
Source: PLoS One. 2020 May 14;15(5):e0233082. doi: 10.1371/journal.pone.0233082 (PMC7224542; doi:10.1371/journal.pone.0233082)
Supplement: S1 Table — (DOCX) [file pone.0233082.s001.docx]

**Overview of diagnostic codes used for exclusion**

| **Obstetric surgery** | **CHOP code** |
| --- | --- |
| Instrumental delivery (forceps, vacuum extractor), breech presentation | 72.x |
| Artificial rupture of amniotic sac | 73.0 |
| Caesarean section and extraction of fetus | 74 |
| Other obstetrical surgery | 75 |
| **Cancer-related diagnosis** | **ICD-10 codes** |
| Malignant neoplasm of vulva | C51.- |
| Malignant neoplasm of vagina | C52.- |
| Malignant neoplasm of cervix uteri | C53.- |
| Malignant neoplasm of corpus uteri | C54.- |
| Malignant neoplasm of uterus, not otherwise specified | C55.- |
| Malignant neoplasm of ovary | C56.- |
| Malignant neoplasm of other not otherwise specified female genital organ | C57.- |
