# Supplementary material for: Regional variation of hysterectomy for benign uterine diseases in Switzerland
Source: PLoS One. 2020 May 14;15(5):e0233082. doi: 10.1371/journal.pone.0233082 (PMC7224542; doi:10.1371/journal.pone.0233082)
Supplement: S2 Table — (DOCX) [file pone.0233082.s002.docx]

**Overview of codes to define comorbidity burden**

|  | ICD 10 code | CHOP code |
| --- | --- | --- |
| Hip fracture | S720, S721, S722 |  |
| Stroke | I63, I64 as 1st or 2nd diagnosis |  |
| Colon cancer | C18, C19 | 457*, 458* or 46* |
| Lung cancer | C34 | 323*, 324*, 325*, 326* or 329* |
| Myocardial infarction | I21 as 1st or 2nd diagnosis |  |

The stars (*) means that it can be followed by anything
