# Supplementary material for: Regional variation of hysterectomy for benign uterine diseases in Switzerland
Source: PLoS One. 2020 May 14;15(5):e0233082. doi: 10.1371/journal.pone.0233082 (PMC7224542; doi:10.1371/journal.pone.0233082)
Supplement: S3 Table — (DOCX) [file pone.0233082.s003.docx]

**Crude- and age-standardized procedure-specific hysterectomy rates**

| **HSA** | **All hysterectomies** | | **Vaginal** | | **Laparoscopic** | | **Abdominal** | |
| --- | --- | --- | --- | --- | --- | --- | --- | --- |
| No. | Crude | Age-std. | Crude | Age-std. | Crude | Age-std. | Crude | Age-std. |
| 48 | 180 | 186 | 120 | 119 | 40 | 45 | 20 | 22 |
| 6 | 205 | 208 | 36 | 35 | 120 | 122 | 49 | 50 |
| 5 | 207 | 208 | 61 | 62 | 62 | 62 | 85 | 85 |
| 4 | 224 | 226 | 49 | 51 | 129 | 128 | 46 | 46 |
| 47 | 225 | 228 | 41 | 37 | 131 | 137 | 53 | 54 |
| 51 | 238 | 238 | 72 | 72 | 117 | 117 | 49 | 49 |
| 46 | 245 | 238 | 85 | 86 | 111 | 106 | 49 | 46 |
| 52 | 242 | 240 | 117 | 117 | 74 | 74 | 50 | 50 |
| 45 | 240 | 244 | 80 | 76 | 111 | 118 | 49 | 50 |
| 8 | 252 | 250 | 57 | 57 | 123 | 121 | 72 | 71 |
| 44 | 247 | 251 | 62 | 59 | 139 | 144 | 46 | 47 |
| 33 | 252 | 252 | 110 | 109 | 82 | 83 | 61 | 61 |
| 54 | 250 | 254 | 122 | 121 | 97 | 101 | 30 | 31 |
| 13 | 255 | 254 | 92 | 90 | 102 | 103 | 59 | 60 |
| 1 | 254 | 255 | 45 | 47 | 175 | 174 | 31 | 31 |
| 31 | 255 | 255 | 70 | 71 | 147 | 148 | 37 | 37 |
| 39 | 245 | 257 | 53 | 48 | 115 | 126 | 77 | 83 |
| 35 | 258 | 258 | 78 | 80 | 123 | 122 | 57 | 57 |
| 53 | 259 | 260 | 62 | 61 | 162 | 163 | 36 | 35 |
| 11 | 257 | 261 | 63 | 60 | 125 | 130 | 69 | 71 |
| 16 | 257 | 263 | 87 | 86 | 83 | 86 | 87 | 90 |
| 29 | 267 | 264 | 127 | 128 | 100 | 98 | 40 | 39 |
| 7 | 266 | 269 | 52 | 52 | 160 | 162 | 53 | 54 |
| 32 | 272 | 273 | 51 | 54 | 184 | 182 | 37 | 37 |
| 49 | 274 | 276 | 101 | 98 | 96 | 100 | 77 | 78 |
| 23 | 279 | 278 | 109 | 107 | 121 | 122 | 49 | 49 |
| 30 | 286 | 285 | 53 | 53 | 206 | 205 | 27 | 27 |
| 3 | 283 | 285 | 108 | 110 | 98 | 97 | 78 | 77 |
| 40 | 294 | 294 | 145 | 147 | 99 | 97 | 50 | 50 |
| 22 | 296 | 295 | 84 | 84 | 129 | 128 | 83 | 82 |
| 2 | 311 | 299 | 94 | 93 | 125 | 118 | 91 | 86 |
| 41 | 316 | 310 | 85 | 85 | 164 | 158 | 68 | 67 |
| 19 | 313 | 316 | 150 | 150 | 97 | 99 | 66 | 68 |
| 26 | 312 | 317 | 128 | 124 | 114 | 119 | 70 | 74 |
| 37 | 319 | 319 | 135 | 130 | 95 | 97 | 90 | 92 |
| 34 | 326 | 321 | 110 | 111 | 153 | 148 | 64 | 62 |
| 21 | 324 | 322 | 85 | 84 | 186 | 185 | 53 | 53 |
| 18 | 322 | 326 | 123 | 118 | 77 | 81 | 123 | 128 |
| 12 | 329 | 334 | 98 | 96 | 157 | 162 | 74 | 76 |
| 20 | 336 | 336 | 71 | 71 | 214 | 214 | 50 | 50 |
| 25 | 342 | 339 | 139 | 145 | 149 | 141 | 54 | 52 |
| 43 | 345 | 343 | 173 | 174 | 124 | 121 | 49 | 48 |
| 38 | 350 | 348 | 140 | 139 | 135 | 135 | 75 | 74 |
| 27 | 353 | 350 | 64 | 64 | 249 | 246 | 40 | 40 |
| 36 | 345 | 351 | 49 | 46 | 266 | 274 | 31 | 31 |
| 42 | 355 | 352 | 73 | 73 | 234 | 231 | 48 | 47 |
| 24 | 368 | 371 | 160 | 168 | 134 | 131 | 74 | 72 |
| 15 | 371 | 376 | 109 | 108 | 175 | 179 | 88 | 90 |
| 50 | 381 | 377 | 179 | 178 | 121 | 119 | 81 | 80 |
| 17 | 374 | 390 | 103 | 100 | 198 | 212 | 74 | 78 |
| 14 | 387 | 393 | 123 | 121 | 163 | 168 | 101 | 104 |
| 10 | 411 | 401 | 103 | 103 | 209 | 202 | 99 | 96 |
| 9 | 420 | 426 | 71 | 71 | 284 | 289 | 65 | 66 |
| 28 | 455 | 456 | 134 | 133 | 152 | 152 | 169 | 171 |

Abbreviations: HSA = Health Service Area; age-std. = age-standardized. Hysterectomy rates/100,000 women/year
