# Supplementary figures and images for: Regional variation of hysterectomy for benign uterine diseases in Switzerland
Source: PLoS One. 2020 May 14;15(5):e0233082. doi: 10.1371/journal.pone.0233082 (PMC7224542; doi:10.1371/journal.pone.0233082)

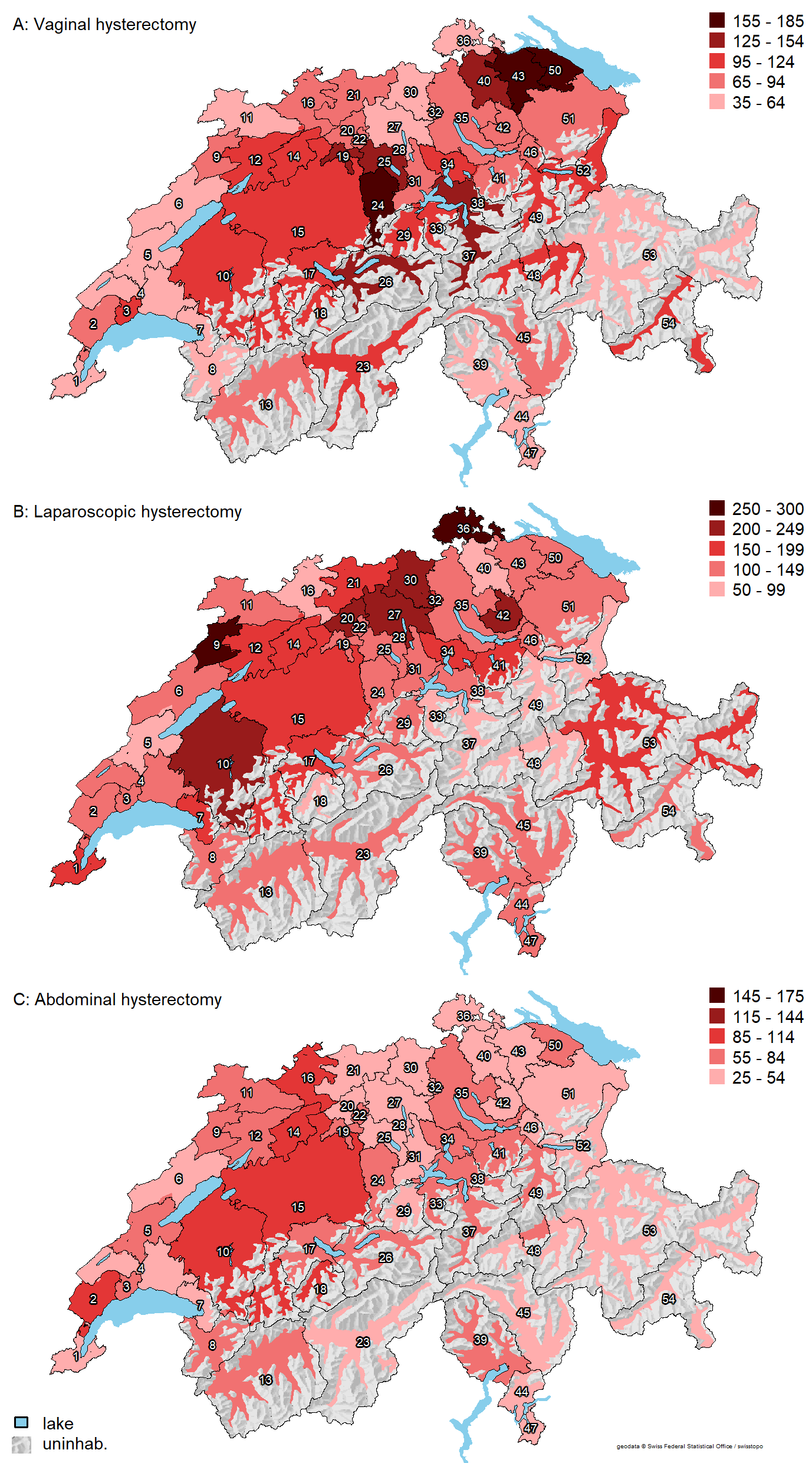

Supplement: S2 Fig — Abbreviations: uninhab. = uninhabited area, HSA = Health Service Area. Hysterectomy rates/100,000 women/per HSA (No. shown within HSA). Reprinted from the Federal Office of Topography swisstopo, Switzerland (https://shop.swisstopo.admin.ch/en/products/maps/overview/relief and shape files derived from postcode-level shape file used to create map of Switzerland, e.g., https://www.geocat.ch/geonetwork/srv/ger/md.viewer#/full_view/973cd117-f1ed-481) under a CC BY license, with permission from Alexandra Frank, original copyright 2006. (TIFF) [file pone.0233082.s005.tiff]
